# cancercelllines.org—a novel resource for genomic variants in cancer cell lines

**DOI:** 10.1093/database/baae030

**Published:** 2024-04-30

**Authors:** Rahel Paloots, Michael Baudis

**Affiliations:** Institute of Molecular Life Sciences, University of Zurich, Winterthurerstrasse 190, Zurich 8057, Switzerland; Swiss Institute of Bioinformatics, Winterthurerstrasse 190, Zurich 8057, Switzerland; Institute of Molecular Life Sciences, University of Zurich, Winterthurerstrasse 190, Zurich 8057, Switzerland; Swiss Institute of Bioinformatics, Winterthurerstrasse 190, Zurich 8057, Switzerland

## Abstract

Cancer cell lines are an important component in biological and medical research, enabling studies of cellular mechanisms as well as the development and testing of pharmaceuticals. Genomic alterations in cancer cell lines are widely studied as models for oncogenetic events and are represented in a wide range of primary resources. We have created a comprehensive, curated knowledge resource—cancercelllines.org—with the aim to enable easy access to genomic profiling data in cancer cell lines, curated from a variety of resources and integrating both copy number and single nucleotide variants data. We have gathered over 5600 copy number profiles as well as single nucleotide variant annotations for 16 000 cell lines and provide these data with mappings to the GRCh38 reference genome. Both genomic variations and associated curated metadata can be queried through the GA4GH Beacon v2 Application Programming Interface (API) and a graphical user interface with extensive data retrieval enabled using GA4GH data schemas under a permissive licensing scheme.

**Database URL**: https://cancercelllines.org

## Introduction

Cancer genomes harbor numerous mutations that can serve in disease classification, clinical prediction as well as to identify targets for therapeutic interventions. These somatic mutations in cancers often occur in genes responsible for DNA replication or repair, enabling uncontrolled proliferation of cancer cells as well as introducing additional mutations to cancer genomes. Single-nucleotide polymorphisms in these genes lead to altered functionality of the gene that is favorable for cancer progression. Another kind of variation often introduced to cancer genomes are structural variants where a large region in the chromosome is altered. Changes to the chromosome where large sequences are deleted or amplified are also referred to as copy number variants (CNVs). Oncogenes are genes endowing cell division functionalities that also contribute to cancer cell progression and additional copies of these genes further enhance cancer growth. Tumor suppressor genes are genes that induce cell death and ensure control mechanisms for correct replication processes, halting cancer progression. In addition to mutations in tumor suppressor genes that alter functionality, these genes are often deleted from cancer genomes. These copy number aberrations lead to deviations from normal diploid state of the genome and are often characteristics for cancer types. For instance, colorectal carcinomas are known to have a duplicated Chromosome 13 that is characteristic for the disease (1–3).

While the molecular analysis of individual tumors provides enormous insights into molecular alterations affecting individual cases or disease types in general, testing of proposed mechanisms and design of pharmacological interventions require the observation of cells of individual tumor types under experimental conditions. These cell lines, representative for specific cancer types and carrying the respective molecular alterations, provide a ubiquitous tool set for foundational and translational cancer research settings. Cancer cell lines are usually obtained by extracting cells from individual tumors and are then cultivated under *in vitro* conditions. For certain applications—e.g. the generation of additional material for molecular testing—tumor-derived cells are only expanded for a few divisions. However, the main application of *in vitro* systems lies in the establishment of immortalized cancer cell lines with the potential for long-term storage and expansion in various experimental settings. Several public and commercial repositories provide thousands of such cell lines for many different cancer types, and some core sets of cell lines (e.g. NCI-60 (4)) are used in most screening systems for the definition of anti-neoplastic pharmaceuticals as well as in studies not directly related to cancer research.

Several databases have been created to collect and characterize cancer cell line data, such as Cellosaurus—a knowledge resource on cell lines (5). While Cellosaurus includes extensive information on cell lines, such as their origin, age at collection, sex, etc., other sources focus on the genomic data of cancer cell lines. Cancer Cell Line Encyclopedia (CCLE) is a database that characterizes cancer cell line mutational data (6). Genomic information relevant to cell lines is also included in curated resources such as ClinVar (7), i.e. information about effects of individual cancer-related mutations which also apply to cell lines. Another example of such resource is Progenetix, a reference resource for sample-specific genomic CNV data of human cancers. In addition to primary cancers, it also includes over 5600 samples of human cancer cell line CNV profiles (8).

Here, we present our novel resource that focuses on cancer cell line variants. We have incorporated and curated data from various resources, including relevant metadata, allowing for numerous query types, e.g. searching by cancer type or variant position. Our resource also provides the unique opportunity to visualize available CNVs and single-nucleotide variants (SNVs) simultaneously.

## Materials and methods

### Variant resources

To create this database, we collected data from the following resources: ClinVar (7), CCLE (6), National Cancer Institute Thesaurus (NCIT) (9), Cellosaurus (5) and Progenetix (8). Specifically, human health-related variants and associated metadata were derived from ClinVar, while known variants from human cancer cell lines were obtained from CCLE. Existing information associated with the cell line and its source originate from Cellosaurus. NCIT cancer classification terms were used to standardize available diagnostic codes and represent diseases hierarchically. Progenetix was the source of our CNV collection and also provided additional publications and geographical information on the cell lines. Moreover, Progenetix provides numerous visualization options particularly for CNVs.

### Variant import and mapping

CNV data for 5754 copy number profiling experiments representing 2163 individual call lines were imported from the Progenetix resource. Data in Progenetix has been generated from various platforms, predominantly genomic array experiments and including a core set of biomedical and technical metadata as described previously (8). SNVs were mapped to Cellosaurus IDs and imported from ClinVar and CCLE resources. As for the CNV data, we used the GRCh38 reference genome and applied a 0-based interbase coordinate system, in accordance with GA4GH recommendations and the GA4GH Variant Representation Specification ((10)). For mutation data derived from the CCLE resource, this process required the translation of the GRCh37 mapped data using a Python-based liftover tool (11).

#### ClinVar

For the addition of cell line–related genomic variants from ClinVar annotations, we retrieved all sequence variation annotations from Cellosaurus. We then translated variants to an appropriate format. For instance, Cellosaurus variant *STK11 p.Gln37Ter (c.109C>T)* was converted to ClinVar format (STK11):c.109C>T (p.Gln37Ter). Identifiers for the variants were then retrieved from (ClinVarVariationRelease_00-latest.xml, last accessed 2023-06-25). Available metadata for each variant were obtained by accessing ClinVar API, for example https://eutils.ncbi.nlm.nih.gov/entrez/eutils/esummary.fcgi?db=clinvar&id=376 334 for the aforementioned variant. Only variant locations for hg38 version were used. Additionally, MyVariant python package (version: 1) was used to add additional HGVS IDs to the variants.

#### CCLE mutations

Cancer cell lines in Cellosaurus dataset include a DepMap ID that is a unique identifier used for cell lines in CCLE data. We used these identifiers to retrieve CCLE mutational data. The mutation dataset was retrieved from CCLE ‘Downloads’ site (12) last accessed: 2022-06-02.

### Cell line metadata

The essential part of the information associated with a cell line originates from Cellosaurus cell line resource (5). Such information includes genome ancestry information, NCIT diagnostic classification of the cell line, age at collection, etc. For the NCIT classification as well as for cell lines, we apply a hierarchical entity representation. For the disease classification, this corresponds to projection of all identified cell line disease codes to the NCIT ‘neoplasm’ tree. For cell line identifiers, we created a physical inheritance hierarchy tree in which derived cell lines are represented as children of the donor cell line and so forth and thereby allow to connect cell line originating from the same donor. Progenetix provides core metadata for CNV samples such as publications and provenance of the sample, enabling the geographical mapping of the samples (13).

### Variant representation

All variants and associated metadata are represented using Beacon v2 default data model (14). To accommodate the requirements of cell line data, some elements of the schemas have been extended. We have used the following ontologies for mapping and standardizing our data: Human Ancestry Ontology (genome ancestry), Sequence Ontology (variant effects), Phenotype And Trait Ontology (genotypic sex), NCIT (histological diagnoses), Uber-anatomy Ontology (anatomic topography), as well as ICD-O 3 for diagnoses (M) and topographic site (T).

### Front-end and API

The cancercelllines.org resource is built from software stack incorporating a MongoDB database back-end, a middleware and API stack from the ‘bycon’ and ‘byconaut’ Python packages and the ‘cancercelllines-web’ React-based Javascript front-end, served through an Apache webserver set-up. Here, the ‘bycon’ software implements a Beacon v2 (14) API and the front-end interacts with this through Beacon conform requests and parsing of the standard Beacon JSON responses. For instance, a search for breast cancer cell lines with genomic variants in the TP53 gene locus would consist of a genomic range query (chromosome, base positions of TP53 start and end coordinates, with optional type or variant base such as ‘SO:0001059’ for a sequence alteration) together with a disease code for breast carcinomas (e.g. ‘NCIT:C4872—Breast Carcinoma’) which is provided as a ‘filter’ to the Beacon API. In our implementation, primary Beacon v2 response will consist of a ‘count’ response for the ‘biosample’ entry type (i.e. provide the number of matched samples) with embedded ‘handover’ links for the retrieval of the individual samples and genomic variations through asynchronous https requests, again using the Beacon v2 protocol.

## Results

### Collection of human cancer cell lines

After mapping of available variants from Progenetix (CNVs), CCLE and ClinVar (SNVs), we created entries for 16 000 unique cell lines from 400 different cancer types according to the NCIT classification. More than 15 000 of these cell lines have mapped variant(s) from ClinVar, over a 1000 cell lines have one or more known SNVs for the cell line from CCLE and more than 2000 cancer cell lines have available CNV profiles processed as part of the Progenetix data pipeline(8). Table 1 shows some of the most common features of donors of all available cell lines in cancercelllines.org. In total, we have data from 4214 donors, of those the genotypic sex is known for 1882 male and 1486 female samples. Among all the samples, the number of male samples exceeds the number of female samples by around 40%. The average age of the donor is 50, with the minimum age being under 1 year old and 94 years old the oldest. Ages of fetal samples were not included. The most frequent cancer type for all mapped cell line samples is melanoma (NCIT:C3224), followed by glioblastoma and lung small cell carcinoma. Interestingly, in all cases except female reproductive system carcinomas, number of male donors per diagnosis is higher than female donors.

**Table 1.** Cancer cell line donor statistics (CNV and SNV samples)

	Sex	Age	Count	Total	Age
Melanoma	Male	54	230	394	53
	Female	51	164		
Glioblastoma	Male	56	87	135	58
	Female	60	48		
Lung small cell carcinoma	Male	57	83	102	57
	Female	56	19		
Colon adenocarcinoma	Male	59	55	99	61
	Female	64	44		
Lung adenocarcinoma	Male	56	60	98	56
	Female	56	38		
Pancreatic ductal adenocarcinoma	Male	60	42	64	61
	Female	64	22		
Adult hepatocellular carcinoma	Male	51	51	59	51
	Female	55	8		
Neuroblastoma	Male	3	33	56	3
	Female	2	23		
Ovarian high-grade serous adenocarcinoma	Female	59	49	49	59
Plasma cell myeloma	Male	59	21	46	61
	Female	62	25		

This table includes samples where genotypic sex and age data are available.

Hierarchical information on cancer cell lines can be found under ‘Cell Line Listings’ on the left. There, the root level of each cell line is shown, and the child levels can be accessed by expanding. The search box in ‘Cell Line Listings’ also allows for hierarchical queries of cell lines. The resulting landing page displays known metadata about the donor of the cell line as well as known parent and child terms.

**Figure 1.**
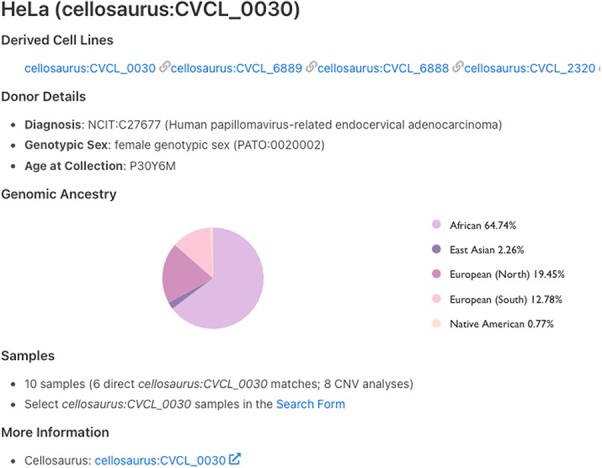
Cell line details page for HeLa. Derived cell lines and information on the cell line donor are listed on this page. The count of associated samples and link to ‘Search Form’ are also shown. The last link on the page redirects to cell line page on Cellosaurus.

Figure 1 illustrates the results for the first human cell line established—HeLa (15). HeLa, a cervical carcinoma cell line, was created in 1951, and the name was derived from the patient’s initials (15, 16). Even today, 70 years later, HeLa is still one of the most widely used cell lines.

All mapped cell lines have a Cellosaurus ID and include metadata such as NCIT disease code associated with cell line as well as genotypic sex of the material and age at collection. Additionally, for some cell lines, genome ancestry data are also available and represented according to the Human Ancestry Ontology model (Fig. 1). CNV frequency plots for the samples of cell line of interest as well as available child terms are shown, followed by mapped SNVs in the annotated variants section.

Moreover, information extraction results for annotated cancer cell line gene information is located under ‘Literature Derived Contextual Information’. More information on this can be found in the study by Smith *et al.* (17).

### Cancer cell line CNV profiles

**Figure 2.**
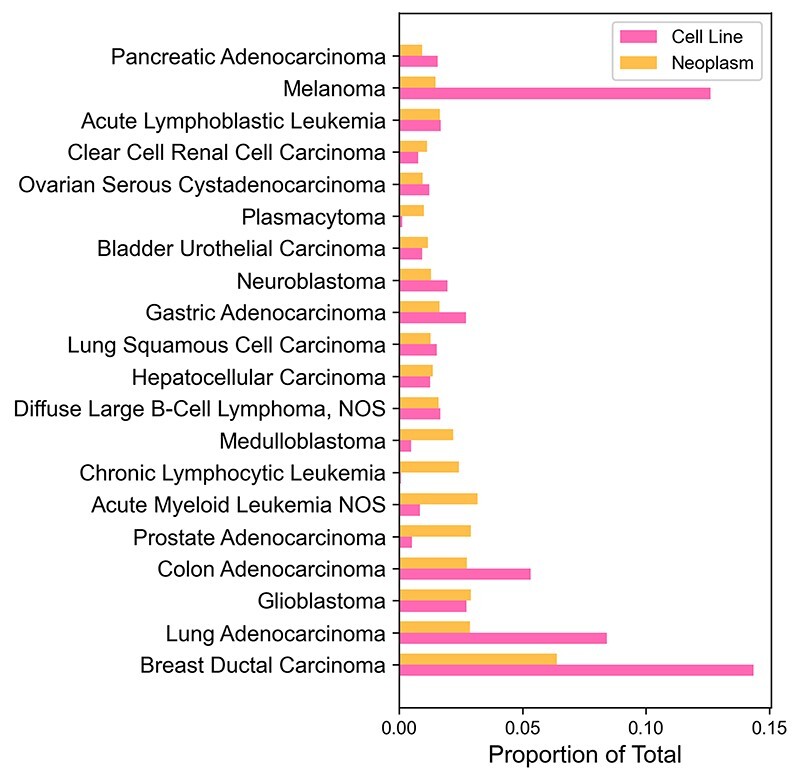
Comparison of copy number sample numbers in cell lines and their origins for the most common cancer types. Twenty most common cancer types (by the number of sample count, excluding ‘Unspecified Tissue’ samples) were picked from Progenetix. Cancer types without any cell lines were excluded as well. Horizontal bars represent the proportion of total sample count for each cancer type.

The CNV data of cancer cell lines originate from Progenetix database where samples related to a cell line have previously been identified from open-source repositories such as Gene Expression Omnibus or from data provided with original publications (8). Figure 2 shows ratios of copy number samples per NCIT diagnostic code of cancer cell lines and their origins in Progenetix. Most frequent cancer type among CNV samples in both cell lines and tumors is ductal breast carcinoma. The large number of breast carcinoma samples could be explained by the large breast cancer detection campaigns that have been implemented worldwide. Unexpectedly, melanomas are underrepresented among primary tumors compared to the sample number in cell lines. A disproportionate number of melanoma samples originates from some studies with a high number of melanoma cell line samples. For example, over 100 cutaneous melanoma samples were retrieved from a comparative study of copy number profiles (18). A well-portrayed origin group is chronic lymphocytic leukemia that is represented by only three cell line samples. One possible explanation could be that slowly progressing cancer types do not acclimate well to *in vitro* environment.

**Figure 3.**
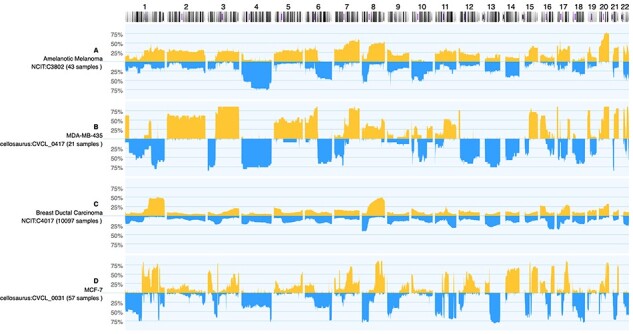
Genomic CNV frequencies comparing cancer-type specific profiles to those from selected cell lines for copy number gains (up) and losses (down; 100%—CNV observed in all samples). While (**A**) and (**C**) display the summary data from 43 amelanotic melanomas (NCIT:C3802) and 10 254 ductal breast carcinomas, respectively, panel (**B**) and (**D**) show summary profiles of cell lines MDA-MB-435 (from 21 instances) and MCF-7 (57 analyses). Although both cell lines were originally classified as ‘breast carcinoma’, the CNV pattern of MDA-MB-435 shows an intriguing similarity to aberrations common in amelanotic melanomas (e.g. +2, +3q, +5, +6p/−6q...). As of note, while the CNV frequency plots are influenced by the expected genomic heterogeneity of tumor samples, the ‘in principle’ expected genomic homogeneity of cell lines (i.e. either 100% or 0%) can be perturbed by genomic instability and leading to inter-sample variations as well as experimental conditions.

It has been shown that cancer cell lines indeed exhibit similar CNV profiles to their origins but have a higher number of mutations (19). Unfortunately, many of the widely used cancer cell lines have been found to be either contaminated or misidentified (20). For instance, cell line MDA-MB-435 was thought to be a breast cancer cell line but was instead found to be originating from melanoma (21). Figure 3 demonstrates cell line MDA-MB-435 compared to ductal breast carcinoma and amelanotic melanoma CNV profiles. By combining the data available in Progenetix and cancercelllines.org, we show that indeed MDA-MB-435 is more similar to amelanotic melanoma.

### SNVs of cancer cell lines

To curate the SNVs in cancer cell lines and their effect on health, we mapped known cell line SNVs to ClinVar variants and pulled cancer cell lines from the CCLE mutations dataset. Table 2 shows the number of resulting variants from ClinVar and CCLE resources. Since ClinVar is a resource for variants related to human health, the number of distinct variants is lower than in CCLE that includes all variants from a set of cell lines. While CCLE only includes around 1000 well-characterized cancer cell lines, known human health-related variants have been found in over 15 000 cancer cell line entities. The most commonly mutated gene in CCLE dataset is TTN, a gene responsible for producing titin protein that is the largest human protein and is a structural sarcomeric protein. TTN mutations have been detected in many cancer types and have been shown to affect tumor mutational burden (22–24). The most common gene in ClinVar dataset is TP53, a tumor suppressor gene that is one of the most frequently mutated genes in cancer (25).

**Table 2.** SNV statistics

	ClinVar	CCLE mutations
Unique variants	1246	23 ,947
Total number of variants	30 960	1 013 244
Number of cell lines	15 750	1292
Most frequent gene	TP53	TTN
Number of genes	144	18 739

### Use cases

#### CNV profiles

Our resource enables finding CNV profiles for cancer cell lines of interest, including the option to automatically include instances of derived cell lines. The query can be performed under ‘Search Cell Lines’ section and subsequently using cell line name or Cellosaurus ID to search. For example, HeLa could be queried by typing ‘HeLa’ or ‘CVCL_0030’ into the ID field. Search query can also be executed by the diagnostic code of the cell line (NCIT) in the ‘Cancer Classification(s)’ field. By default, all child terms found for the cell line (or diagnostic code) will be included. This can be changed under ‘Include Child Terms’ field. The resulting landing page will show resulting CNV frequency plot with options to list existing biosamples, to see where these samples were from geographically and also to list the known annotated variants for these cell lines. Additional visualization options can be found under ‘Visualization options’. In addition to CNV frequency plots, this enables clustered view of the samples.

#### SNV data

**Figure 4.**
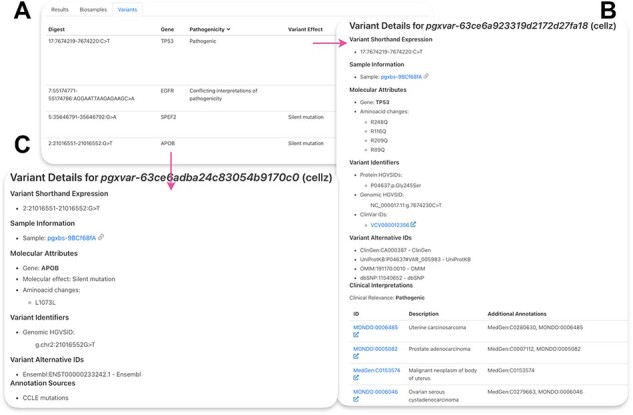
Lung adenocarcinoma cell line PC-9 SNVs. (**A**) Table of resulting variants for PC-9. ‘Digest’ shows the genomic location and the affected nucleotides of the variant, ‘Gene’—the affected gene, ‘Pathogenicity’—reported effect of the variant on human health, ‘Variant Effect’—effect of the variant on the gene product. (**B**) Example of a ClinVar variant. (**C**) Example of a CCLE mutation.

To access our SNV data, cell line on interest can be queried in ‘Search Cell Lines’ like for CNV samples but additional field needs to be entered under Query by Position, Variant Type: SO:0001059 (any sequence alteration—SNV, insertion-deletion). The resulting matched SNVs can be found under ‘Variants’ section (Figure 4A). There, variants are listed and can be sorted by the field of interest. ‘Digest’ shows the genomic location and the affected nucleotides of the variant, and ‘Gene’ represents the affected gene. Pathogenicity refers to known clinical impact of the variant from ClinVar annotations. Variant effect shows the effect of the mutation, according to sequence ontology.

Clicking on the variant ID leads to variant page (Figure 4B and 4C). Figure 4B shows the results for a variant in TP53 from ClinVar. Available variant HGVS identifiers, ClinVar identifiers as well as alternative IDs for this variant from other resources are shown. Under ‘Clinical Interpretations’, disease ontologies are listed by ID and descriptions are provided on the left. Clicking on the ID of interest will redirect to the disease ontology page. Figure 4C shows available mutation data for a variant in the APOB gene. ClinVar is a database for phenotypic health-related variants; therefore, each variant includes more information compared to CCLE that only shows cancer cell line–specific variant information. Information about the molecular attributes of this mutations can be found for CCLE variants such as amino acid changes and molecular effect. Some genomic HGVS identifiers are also available for CCLE variants. More information is included in a detailed user guide (Supplementary Materials).

## Discussion

Cancer cell lines are important model systems in many areas of biomedical research. While the knowledge about their genomic variations represents an essential component for their effective and accurate utilization, this information is dispersed in different types of databases and repositories. Here, we have presented cancercelllines.org, a website and knowledge resource with a comprehensive collection of curated cancer cell line genome variants. In this database, we have included a large collection of annotated sequence variants and generated copy number profiling data as well as curated metadata including identifier-based links to external donor repositories and information resources. Importantly, cell line entities are linked hierarchically according to their provenance thereby facilitating analyses of mutational dynamics as well help with the identification of labeling inconsistencies. While various excellent resources such as COSMIC (26) and CCLE (6) contain data about genomic variants in cancer cell lines, our resource offers a unique, comprehensive functionality to assess genomic data combined from various resources, including a large, unique set of genome-wide CNV profiling data.

Cancer cell lines can be used in different fields in life sciences but predominantly serve to study disease mechanisms of cancers and evaluate potential targets of therapeutic interference. The use of these model systems in conjunction with genome profiling data from native tumor samples can be advantageous to select cell lines for *in vitro* experiments matching the tumor types of interest, potentially beyond the confinements of diagnostic classifications. The comparison of CNV profiling data between cancer cell lines and native tumor samples may provide a new avenue for the use of cancer cell line models in ‘matched genomics’ scenarios. The integration of cancer cell line data with data from the Progenetix resource—facilitated through common frameworks, annotation standards, query and visualization methods—enables both the visual identification of similarities in data patterns (cf. Figure 3) and the retrieval of standardized data for offline analyses.

Data discovery and retrieval in cancercelllines.org is enabled through the use of the ‘Beacon’ API, a standard of the Global Alliance for Genomics and Health, and associated schemas for genomic as well as biomedical and technical metadata. Importantly, the support of these standards in an open access data setting allows the integration of cancercelllines.org into federated data discovery scenarios (27), where each resource provides complementary data under a common access protocol.

## Supplementary Material

baae030_Supp

## Data Availability

All data on cancercelllines.org can be accessed through API or by downloading files of interest. We have provided the following supplementary files: User guide (PDF). User guide can also be found here: https://docs.cancercelllines.org/user-guide/.
