# Supplementary material for: cancercelllines.org—a novel resource for genomic variants in cancer cell lines
Source: Database (Oxford). 2024 Apr 30;2024:baae030. doi: 10.1093/database/baae030 (PMC11060486; doi:10.1093/database/baae030)
Supplement: baae030_Supp [file baae030_supp.zip › suppl_data/user_guide.pdf]

User Guide

General

On the front page you can find some background information on our database and a randomly created cancer cell line CNV frequency plot where amplifications are shown in yellow and deletions in blue. The left panel shows different query options.

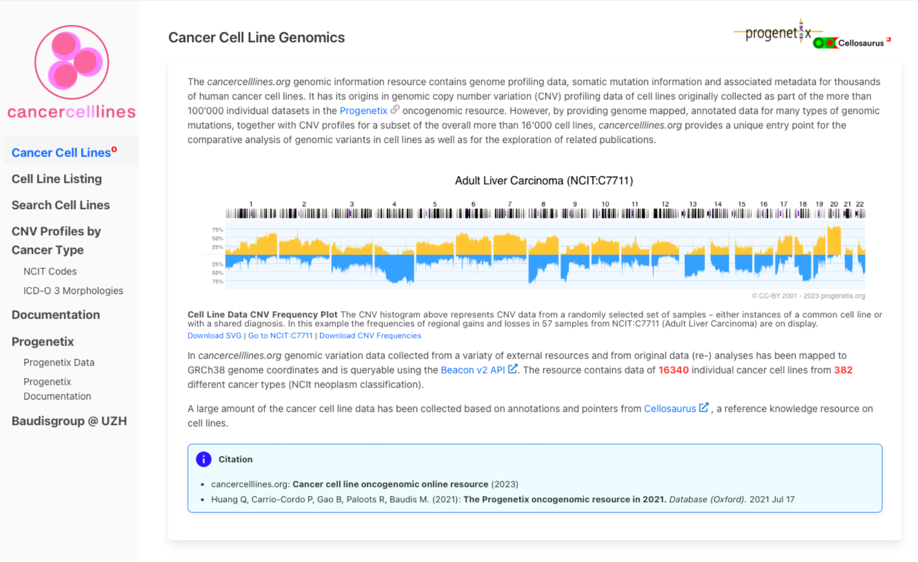

Cell Line Listings - find all available cancer cell lines represented hierarchically. Search box allows for the query of cell line of interest. Returns info on cell line origins, hierarchies, variants and cell line gene information extraction. **Search Cell Lines** - general search box for variant queries, cell line specific queries and queries by cancer type. **CNV profiles by Cancer Type** - get cancer cell line CNV profiles for cancer type of interest. Get hierarchical cancer types by NCIT or ICDO classifications.

Cell Lines Listing

Search Form

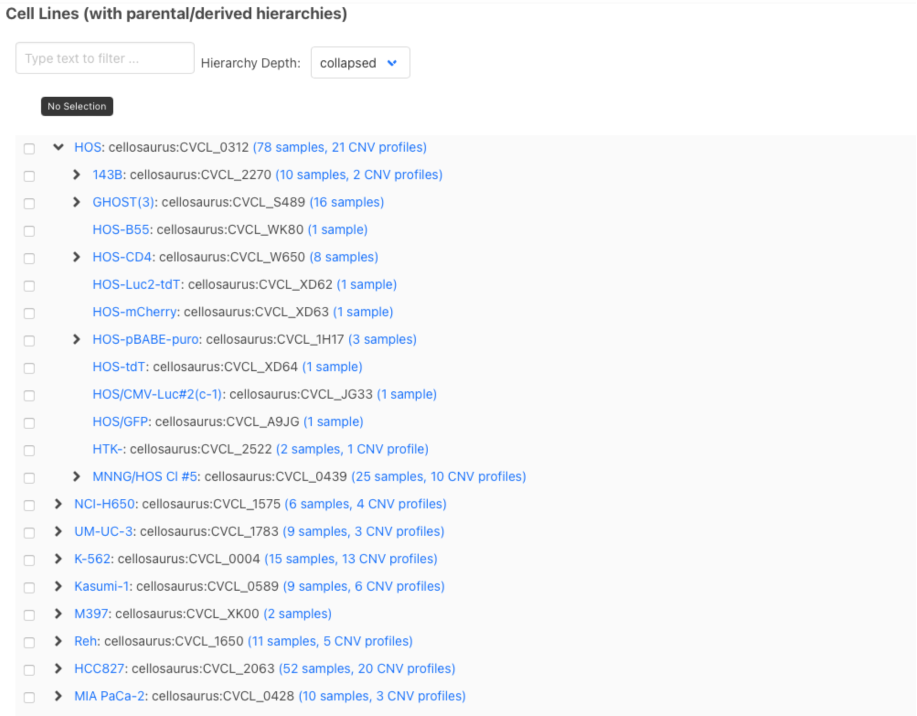

On “Cell Lines Listing” page, available cancer cell lines are listed and child terms can be expanded by clicking on the “>”. You can also search for a cell line either by name or cellosaurus ID in the search box. Numbers indicate available biosamples and CNV profiles available for cell line of interest. By clicking on cell line you will be directed to cell line results page.

Cell Line Page

Cell line results page first gives information on the cell line. First, derived and parental cell lines are listed. Then information on the donor individual is listed including diagnosis, sex and age. Genomic ancestry is also available for a selection of cell lines. Below is a section on sample numbers and further search options. A link to Cellosaurus cell line knowledge resource is also provided for more information.

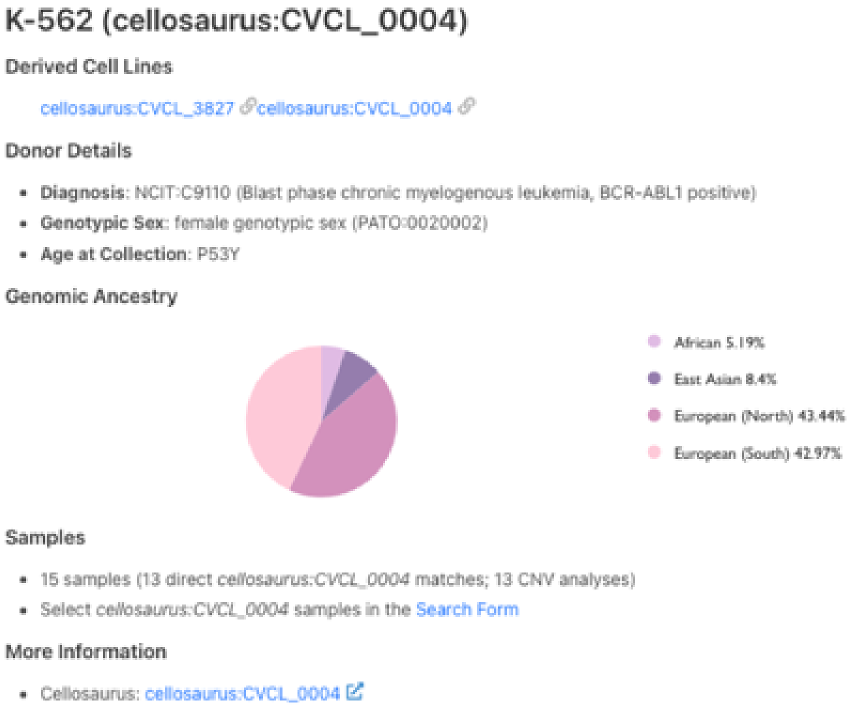

Figure 1: Cell line page

Annotated Variants for cellosaurus:CVCL_0004

Digest	Gene ^	Pathogenicity	Variant Effect	Variant Instances
17:68932401-68932403:->A	ABCA8		Frameshift variant	V: pgxvar-63ce6acda24c83054b8bbc02<br>B: pgxbs-c43B6e8E
7:48278880-48278881:C>A	ABCA13		Missense variant	V: pgxvar-63ce6acda24c83054b8bbcd3<br>B: pgxbs-c43B6e8E
7:87409347-87409348:G>A	ABCB4		Missense variant	V: pgxvar-63ce6acda24c83054b8bbcd7<br>B: pgxbs-c43B6e8E
11:34356773-34356774:G>T	ABTB2		Silent mutation	V: pgxvar-63ce6acda24c83054b8bbb90<br>B: pgxbs-c43B6e8E
2:210205750-210205751:C>A	ACADL		Missense variant	V: pgxvar-63ce6acda24c83054b8bbc5e<br>B: pgxbs-c43B6e8E
5:33534833-33534834:G>C	ADAMTS12		Splice region variant	V: pgxvar-63ce6acda24c83054b8bbca0<br>B: pgxbs-c43B6e8E
11:130449684-130449685:C>A	ADAMTS15		Missense variant	V: pgxvar-63ce6acda24c83054b8bbba8<br>B: pgxbs-c43B6e8E
5:5209159-5209161:->A	ADAMTS16		Frameshift variant	V: pgxvar-63ce6acda24c83054b8bbca1<br>B: pgxbs-c43B6e8E
6:143433897-143433898:A>C	ADAT2		Missense variant	V: pgxvar-63ce6acda24c83054b8bbcda<br>B: pgxbs-c43B6e8E
4:7773002-7773003:C>A	AFAP1		Missense variant	V: pgxvar-63ce6acda24c83054b8bbc90<br>B: pgxbs-c43B6e8E

« ‹ › » Page **1 of 48**

The following section shows annotated single nucleotide variants for the cell line. Variants can be ordered by gene, for example, by clicking "Gene" on the table header. Variants shown in this section include derived variants from parental cell lines.

Subset CNV Frequencies

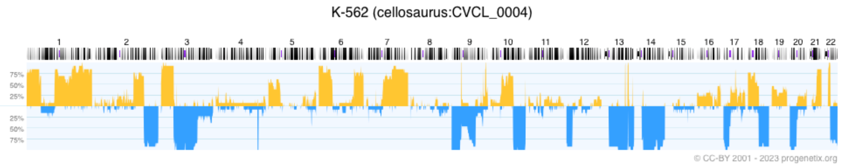

Download SVG | Go to cellosaurus:CVCL_0004 | Download CNV Frequencies

Subset CNV frequencies are visualized in the next section. Links below frequency plot direct to downloading the plot in SVG format as well as to the search page.

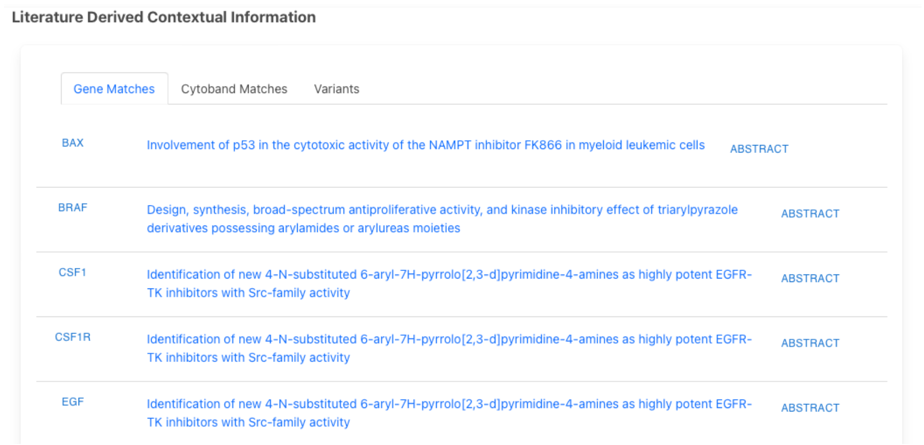

The last section of the cell line results page shows literature derived gene information on the cell lines. Click on the gene name to visualize it on the frequency plot. Clicking on the article title redirects you to the article page on PubMed. Abstract of the paper will be expanded upon left-click.

Search Cell Lines

You can also search for individual cell line by the name or ID or search for cell lines that match the diagnosis of interest (eg NCIT code) or search for all cell lines that harbour a specific variant.

All search form fields: * The top row of the search fields offers some example queries, for both cell lines and positional queries. * You can use “Gene Spans” and “Cytobands” buttons to limit your results to a range of a gene of interest or a specific cytoband. * Default dataset is Cancer Cell Lines Collection but Progenetix collection is also available to enable queries with tumor samples * Gene Symbol - search for a variant in a gene * Query by Position - show fields to enable search by position * Chromosome - enter chromosome of interest. Refseq IDs are also accepted. * Variant Type - specify variant type by EFO term. This field can be used to receive available SNVs. * Start or Position - start of sequence variation of 1-based genomic positions * End - end of sequence variation of 1-based genomic positions * Minimum/Maximum variant length - for queries with pre-defined variant lengths * Reference/Alternate Base(s) - enter reference and altered nucleotide bases * Cell Line IDs/Names - this field can be used to query cell lines by their IDs. Cellosaurus IDs or cell line names (Cellosaurus nomenclature) are accepted as input for cell lines. * Cancer Classification(s) - use NCIT or ICDO disease codes to search for cell lines of the diagnostic code. * Genotypic Sex - search for male or female cell line samples. * Filtering options - additional filters to limit the number of search result, define filter logic and include child terms. * Filters - option to add additional free comma separated filters e.g. PMID, CVCL etc. * Filter logic - and/or * Include Child Terms - includes available child terms or only shows exact matches. Can be applied for

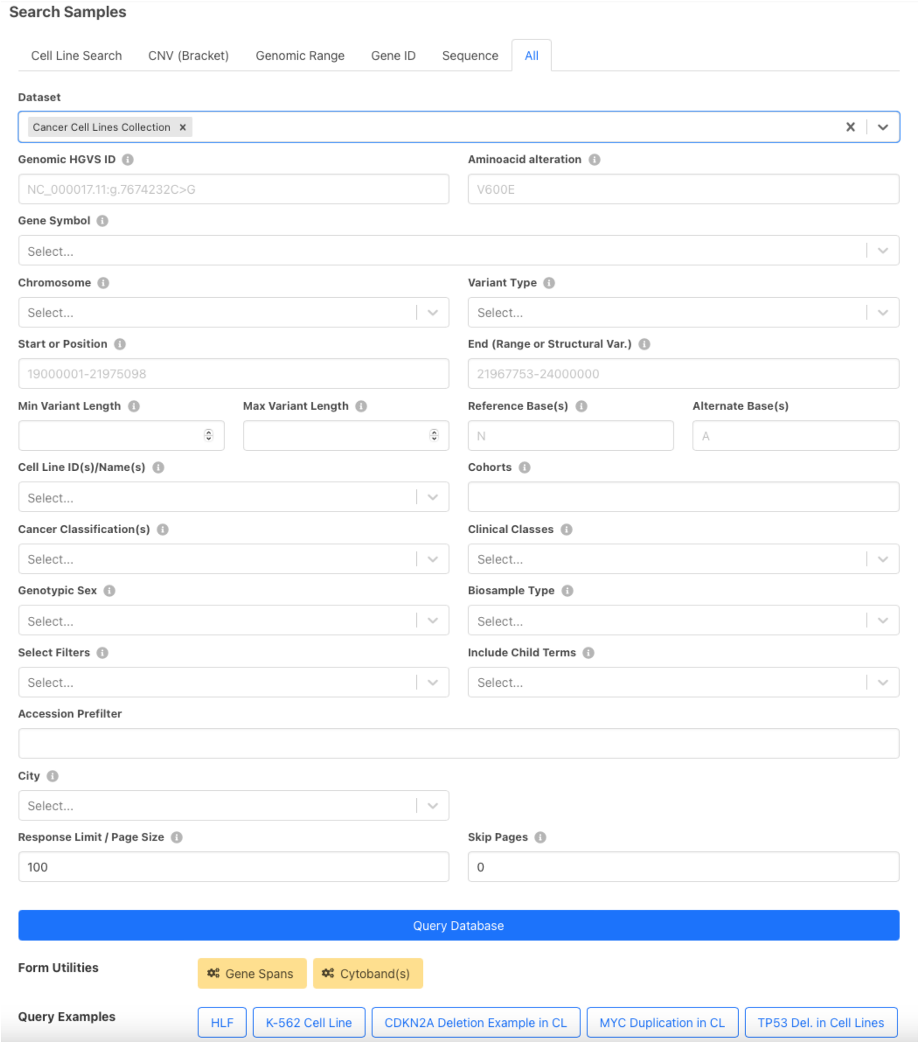

Figure 2: Search form

cell lines and diagnostic codes as well. * Response Limit/Page Size - default is 1000, can be increased or decreased * Skip Pages - select number of pages to be skipped * City - search samples by the annotated city

The top panel includes 5 other short query types: Cell Line Search, CNV query, Genomic Range, Gene ID and Sequence

Search Results: Cell Line Query

Here are the search results for an example query listed in the search form - K-562 cell line. All data in our database is uses GRCh38 assembly version. Used filters, in this case Cellosaurus ID for K-562 are shown on top. Cellz - an internal short name for the cancer cell lines dataset. Dataset responses can be viewed in a JSON format. In the results tab, the frequency plot for query result is shown. On the last page, you can find a table of matched subsets and sample numbers. These data can also be downloaded in TSV or JSON format.

Biosamples

Clicking on "Biosample ID" on the Biosamples tab will lead to the sample page. There info about the cell line origin is shown - same as in Cell Line Page. Additional reference for the sample are provided and CNV plot of the sample is shown (when available).

Visualization options land on a separate page with advanced options for data visualization. In addition to frequency plot, a sample plot with all clustered individual plots is shown.

Biosamples tab lists samples for this query. Associated diagnostic codes are also shown. Identifiers column shows other known sample identifiers such as GEO IDs or PMID.

We have geographical info about where the sample was processed. That can be visualized in the Biosamples Map tab. This info is only available for CNV samples.

Search Results: SNV query

To access our SNV data, cell line on interest can be queried in "Search Cell Lines" like for CNV samples but additional field needs to be entered under Query by Position -> Variant Type -> SO:0001059 (any sequence alteration - SNV, INDEL…). This yields in all available SNVs for the cell line of interest. For example, search results for PC-9 cell line SNVs (Filtering Options -> only match exact terms) result in:

CNV plot is empty in this case as no CNV samples were included in the query. Variants are listed under "Variants" tab. Here, a small part of matched variants is listed. The results can be sorted by Digest, Gene, Pathogenicity or Variant

Sample Details for *pgxbs-kftvipwj*

Description

Chronic myelogenous leukemia [cell line K-562]

Diagnostic Classifications

- Blast phase chronic myelogenous leukemia, BCR-ABL1 positive: NCIT:C9110
- Chronic myelogenous leukemia, BCR/ABL positive: pgx:icdom-98753
- Bone marrow: pgx:icdot-C42.1
- bone marrow: UBERON:0002371

Cell Line Info

- Instance of cellosaurus:CVCL_0004

Donor Details

- **Diagnosis**: NCIT:C9110 (Blast phase chronic myelogenous leukemia, BCR-ABL1 positive)
- **Genotypic Sex**: female genotypic sex (PATO:0020002)
- **Age at Collection**: P53Y

Genomic Ancestry

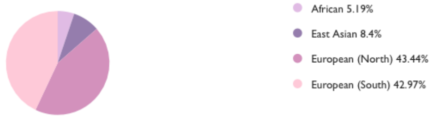

Provenance

- Origin: Boston, United States

External References

- Garraway LA, Widlund HR et al. (2005): Integrative genomic analyses identify MITF as...: PMID:16001072
- K-562: cellosaurus:CVCL_0004
- K-562: geo:GSM50195
- Affymetrix 100K SNP array data-: geo:GSE2520
- Affymetrix GeneChip Human Mapping 50K Hind - prerelease: geo:GPL2014

CNV Plot

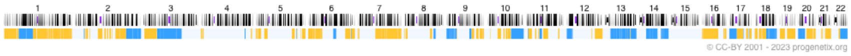

Download

- Sample data as Beacon JSON
- Sample data as Beacon Phenopacket JSON
- Sample variants as Beacon JSON
- Sample variants as Progenetix .pgxseg file
- Sample variants as (experimental) VCF 4.4 file

Raw Data

⇒ click to show/hide

Figure 3: Sample details

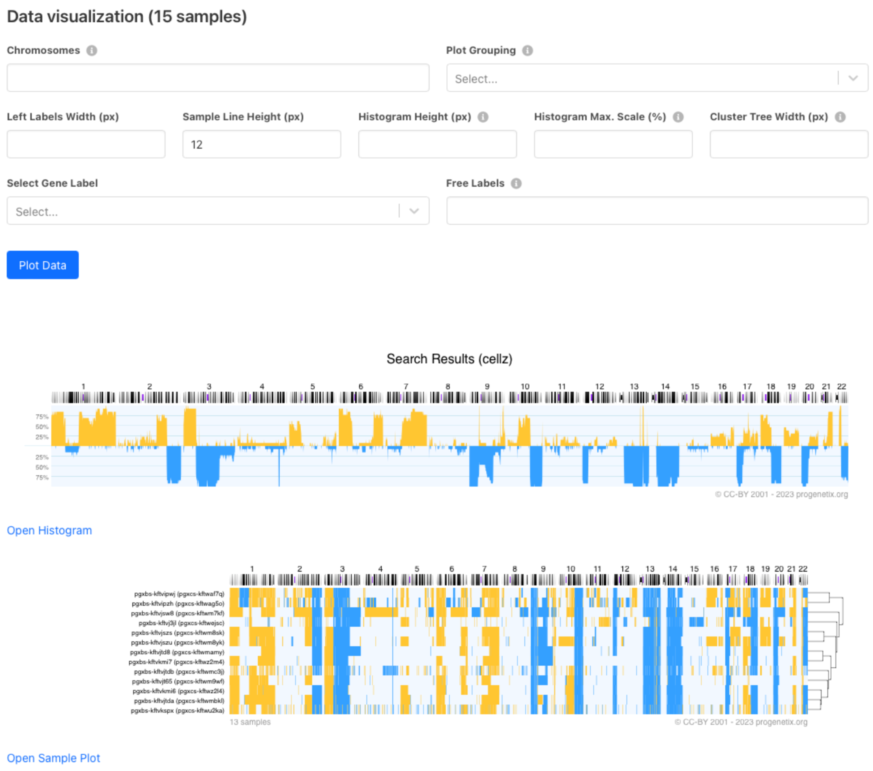

Figure 4: Visualization options

Results Biosamples Biosamples Map

Biosample Id	Dx Classifications	Identifiers
pgxbs-c43B6e8E	NCIT:C9110 Blast phase chronic myelogenous leukemia, BCR-ABL1 positive<br>pgx:icdom-98753 Chronic myelogenous leukemia, BCR/ABL positive<br>pgx:icdot-C42.1 Bone marrow<br>UBERON:0002371 bone marrow	cellosaurus:CVCL_0004 K-562
pgxbs-6B0f6761	NCIT:C9110 Blast phase chronic myelogenous leukemia, BCR-ABL1 positive<br>pgx:icdom-98753 Chronic myelogenous leukemia, BCR/ABL positive<br>pgx:icdot-C42.1 Bone marrow<br>UBERON:0002371 bone marrow	cellosaurus:CVCL_3827 K562/Adr
pgxbs-kftvipwj	NCIT:C9110 Blast phase chronic myelogenous leukemia, BCR-ABL1 positive<br>pgx:icdom-98753 Chronic myelogenous leukemia, BCR/ABL positive<br>pgx:icdot-C42.1 Bone marrow<br>UBERON:0002371 bone marrow	PMID:16001072 Garraway LA, Widlund HR et al. (2005): Integrative genomic analyses identify MITF as...<br>cellosaurus:CVCL_0004 K-562<br>geo:GSM50195<br>geo:GSE2520<br>geo:GPL2014
pgxbs-kftvipzh	NCIT:C9110 Blast phase chronic myelogenous leukemia, BCR-ABL1 positive<br>pgx:icdom-98753 Chronic myelogenous leukemia, BCR/ABL positive<br>pgx:icdot-C42.1 Bone marrow<br>UBERON:0002371 bone marrow	PMID:16001072 Garraway LA, Widlund HR et al. (2005): Integrative genomic analyses identify MITF as...<br>cellosaurus:CVCL_0004 K-562<br>geo:GSM50259<br>geo:GSE2520<br>geo:GPL2015
pgxbs-kftvj3jl	NCIT:C9110 Blast phase chronic myelogenous leukemia, BCR-ABL1 positive<br>pgx:icdom-98753 Chronic myelogenous leukemia, BCR/ABL positive<br>pgx:icdot-C42.1 Bone marrow<br>UBERON:0002371 bone marrow	PMID:20215515 Rothenberg SM, Mohapatra G et al. (2010): A genome-wide screen for microdeletions reveals...<br>cellosaurus:CVCL_3827 K562/Adr<br>geo:GSM827221<br>geo:GSE20306<br>geo:GPL3720

Figure 5: Biosamples table

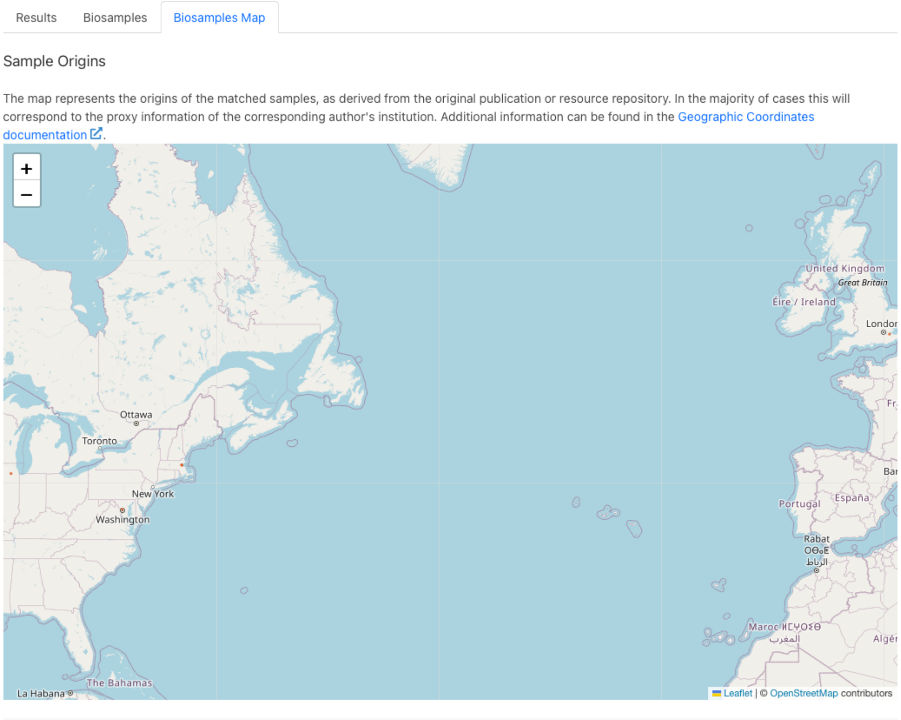

Figure 6: Map of biosamples

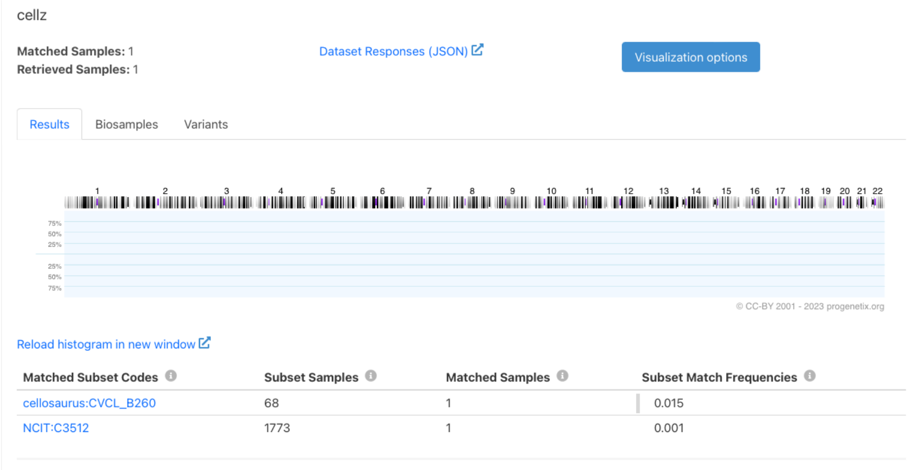

Figure 7: SNV results

Effect by clicking on the column name. Click on the variant ID in blue for more information on the variant.

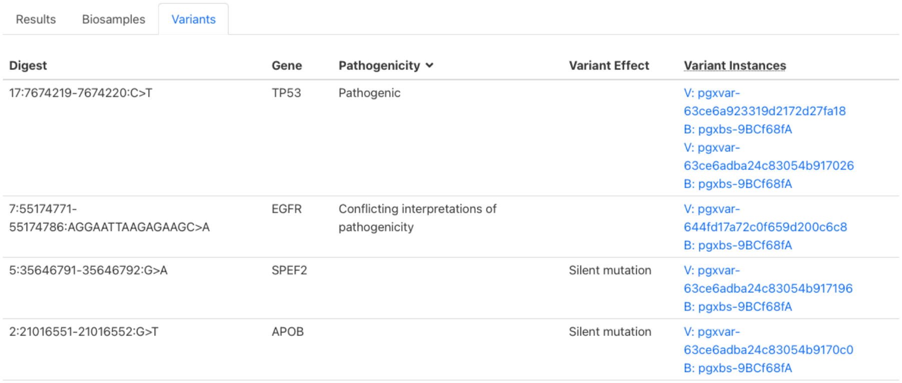

Results | Biosamples | Variants

Digest	Gene	Pathogenicity	Variant Effect	Variant Instances
17:7674219-7674220:C>T	TP53	Pathogenic		V: pgxvar-63ce6a923319d2172d27fa18<br>B: pgxbs-9BCf68fA<br>V: pgxvar-63ce6adba24c83054b917026<br>B: pgxbs-9BCf68fA
7:55174771-55174786:AGGAATTAAGAGAAGC>A	EGFR	Conflicting interpretations of pathogenicity		V: pgxvar-644fd17a72c0f659d200c6c8<br>B: pgxbs-9BCf68fA
5:35646791-35646792:G>A	SPEF2		Silent mutation	V: pgxvar-63ce6adba24c83054b917196<br>B: pgxbs-9BCf68fA
2:21016551-21016552:G>T	APOB		Silent mutation	V: pgxvar-63ce6adba24c83054b9170c0<br>B: pgxbs-9BCf68fA

Figure 8: Variants table

The example below shows available data for a variants collected from CCLE mutation set.

Here is an example for a matched variant in ClinVar dataset:

CNV Profiles by Cancer Type

Like in Cell Line Listings, diagnostic codes are organized hierarchically. We provide NCIT and ICDO diagnostic codes for cancer cell lines. Number of biosamples and CNV profiles per NCI term are indicated. Clicking on available samples will lead to search form that can be used as described above.

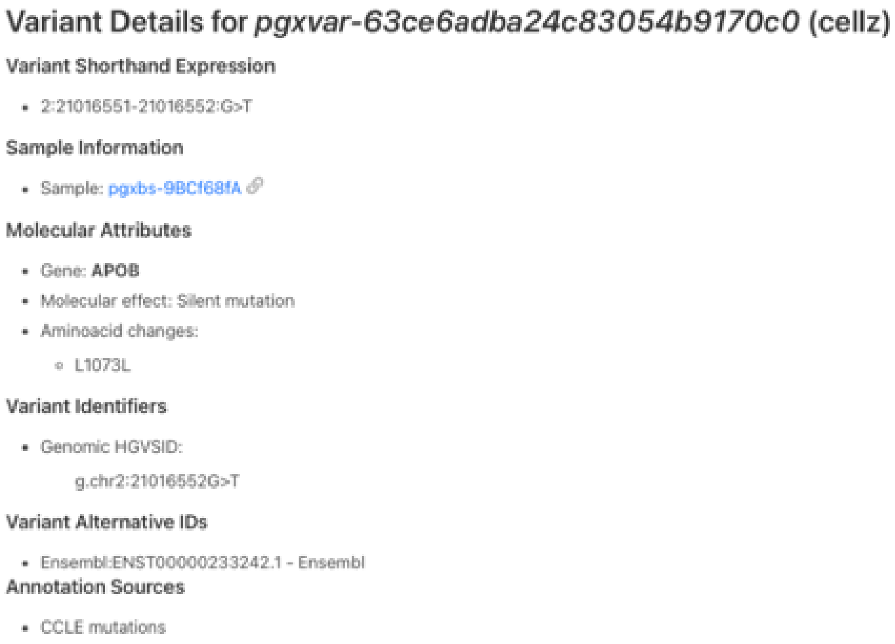
Variant Details for *pgxvar-63ce6adba24c83054b9170c0* (cellz)

Variant Shorthand Expression

- 2:21016551-21016552:G>T

Sample Information

- Sample: pgxbs-9BCf68fA

Molecular Attributes

- Gene: **APOB**
- Molecular effect: Silent mutation
- Aminoacid changes:
  - L1073L

Variant Identifiers

- Genomic HGVSID:
  g.chr2:21016552G>T

Variant Alternative IDs

- Ensembl:ENST00000233242.1 - Ensembl

Annotation Sources

- CCLE mutations

Figure 9: Variant details CCLE

Variant Details for *pgxvar-63ce6a923319d2172d27fa18* (cellz)

Variant Shorthand Expression

- 17:7674219-7674220:C>T

Sample Information

- Sample: pgxbs-9BCf68fA

Molecular Attributes

- Gene: **TP53**
- Aminoacid changes:
  - R248Q
  - R116Q
  - R209Q
  - R89Q

Variant Identifiers

- Protein HGVSIDs:
  - P04637:p.Gly245Ser
- Genomic HGVSID:
  NC_000017.11:g.7674230C>T
- ClinVar IDs:
  - VCV000012356

Variant Alternative IDs

- ClinGen:CA000387 - ClinGen
- UniProtKB:P04637#VAR_005983 - UniProtKB
- OMIM:191170.0010 - OMIM
- dbSNP:11540652 - dbSNP

Clinical Interpretations

Clinical Relevance: **Pathogenic**

ID	Description	Additional Annotations
MONDO:0006485	Uterine carcinosarcoma	MedGen:C0280630, MONDO:0006485
MONDO:0005082	Prostate adenocarcinoma	MedGen:C0007112, MONDO:0005082
MedGen:C0153574	Malignant neoplasm of body of uterus	MedGen:C0153574
MONDO:0006046	Ovarian serous cystadenocarcinoma	MedGen:C0279663, MONDO:0006046

Figure 10: Variant details ClinVar
